# Mechanism of KLF4 Inhibition of epithelial-mesenchymal transition in gastric cancer cells

**DOI:** 10.1515/biol-2025-1247

**Published:** 2025-12-31

**Authors:** Yue Fu, Ze Lu, Xia Liu, Chunming Li

**Affiliations:** Department of Histology and Embryology, School of Basic Medicine, Zunyi Medical University, Zunyi, P.R. China; Zunyi Medical University, Zunyi, Guizhou 563003, P.R. China; Department of Pathology, Banan Hospital Affiliated to Chongqing Medical University, Chongqing, P.R. China

**Keywords:** gastric cancer cells, KLF4, EMT, Wnt/β-catenin signaling pathway, XAV-939, SKL2001

## Abstract

This study investigates the mechanism by which Krüppel-like Factor 4 (KLF4) suppresses epithelial-mesenchymal transition (EMT) in gastric cancer cells. Using Western blot (WB) and reverse transcription-quantitative PCR (RT-qPCR), we evaluated KLF4 protein and mRNA expression levels across gastric cancer cell lines with varying degrees of differentiation. The BGC-823 cell line, which exhibited the lowest KLF4 expression at both protein and mRNA levels, was selected for transfection with a KLF4-overexpressing lentivirus. Following transfection, the Wnt signaling pathway inhibitor XAV-939 and agonist SKL2001 were administered to the KLF4-overexpressing cells. Subsequent Western blot and RT-qPCR analyses were performed to assess the expression of Wnt signaling components and EMT-related markers. Results demonstrated that KLF4 overexpression inhibits EMT in gastric cancer cells through the Wnt/β-catenin signaling pathway. Thus, this study concludes that KLF4 may modulate EMT in gastric cancer cells via the Wnt/β-catenin pathway.

## 1 Introduction

Gastric cancer (GC) is a major global health challenge, with an estimated annual incidence of 990,000 cases and approximately 738,000 fatalities worldwide [1]. Dietary habits are significant risk factors for GC. In particular, processed foods, red and processed meats, alcohol, and diets high in salt, fat, and cholesterol elevate the risk [2]. Given the crucial roles of microbes and chronic inflammation in GC pathogenesis highlighted by recent research, it is imperative to further investigate their underlying mechanisms [3]. The prognosis for intermediate and advanced GC remains poor, with limited therapeutic options. Consequently, chemotherapy, targeted therapy, and immunotherapy have become the cornerstone of treatment for these disease stages [4]. Nevertheless, the efficacy of these treatments is often limited by drug resistance, thus highlighting the critical need to identify new therapeutic targets for novel GC treatment strategies.

Krüppel-like Factor 4 (KLF4) is a critical transcription factor that maps to chromosome 9q31 and encodes a protein consisting of 513 amino acids [5]. KLF4 is implicated in the pathogenesis of a spectrum of malignancies, including non-small cell lung cancer, gastric cancer, hepatocellular carcinoma, pancreatic cancer, renal cell carcinoma, and breast cancer [6], [7], [8], [9], [10], [11], [12]. While Sreeparna et al. [5] have investigated KLF4’s functions and regulatory mechanisms across various cancers, identifying it as a potential therapeutic target and clinical biomarker (particularly in pancreatic cancer), its mechanism of action in GC remains unclear.

Epithelial-mesenchymal transition (EMT) is a dynamic and reversible process characterized by the transformation of cells from an epithelial to a mesenchymal phenotype [13], 14]. EMT is broadly classified into three types: Type 1, which is associated with embryonic and organ development; Type 2, involved in wound healing, tissue regeneration, and organ fibrosis; and Type 3, which is linked to cancer cell migration [15], 16]. Raghu et al. [17] demonstrated that KLF4 can delay the onset of EMT by suppressing multiple EMT transcription factors, thereby revealing its pivotal regulatory role in this process.

The canonical Wnt/β-catenin signaling pathway is characterized by the tight regulation of cytoplasmic β-catenin, a central mediator of this pathway [18]. The Wnt/β-catenin signaling pathway plays a pivotal role in promoting the proliferation, migration, and invasion of various cancers, including gastric, lung, and liver cancer. [19], [20], [21]. Extensive evidence indicates that inhibiting the Wnt/β-catenin signaling pathway effectively suppresses EMT [22], 23]. This study examines if KLF4, as a transcription factor, regulates EMT in gastric cancer cells via the Wnt/β-catenin signaling pathway.

## 2 Materials and methods

*Cells and Cell Culture*. Cell Lines and Culture: The following human gastric adenocarcinoma cell lines were used: MKN-28 (highly differentiated), SGC-7901 (moderately differentiated), and BGC-823 (poorly differentiated). Cells were cultured in complete medium (RPMI-1640 with 10 % FBS and 1 % penicillin-streptomycin) at 37 °C and 5 % CO_2_, with medium changes every two days.

*Lentiviral Transfection and Stable Line Construction*. Following seeding in 24-well plates (5 × 10ˆ4 cells/well), BGC-823 cells were infected with lentivirus at 30 % confluency. The groups included: BGC823-Blank (uninfected), BGC823-Control (empty vector), and BGC823-OE_KLF4_ (KLF4-overexpressing lentivirus from Shanghai He Yuan Biologicals). Using an optimized MOI of 10 and 5 μg/ml Polybrene, the virus-containing medium was added. After 12 h, this medium was replaced with fresh medium. Fluorescence microscopy at 72 h confirmed infection efficiency, after which successful transductants were selected with 5 μg/ml puromycin for downstream applications.

*Reverse Transcription-Quantitative PCR (RT-qPCR)*. Total RNA was extracted from cells using RNAiso (Takara) reagent (1 mL per vial) followed by incubation on ice for complete lysis. The lysate was centrifuged at 12,000×*g* for 20 min at 4 °C. The resulting supernatant was mixed with 200 μL of chloroform, vigorously shaken, and incubated for 5 min. After centrifugation at 12,000×*g* for 15 min at 4 °C, the aqueous phase was collected and combined with 500 μL of isopropanol. The mixture was inverted thoroughly, incubated for 10 min, and centrifuged again (12,000×*g*, 15 min, 4 °C) to pellet the RNA. The pellet was washed with 500 μL of 75 % ethanol, centrifuged (12,000×*g*, 5 min, 4 °C), and air-dried for 5 min with the tube lid open. Finally, the RNA was dissolved in 60 μL of DEPC-treated water. RNA concentration and purity were determined using a spectrophotometer. cDNA was synthesized on ice using a reverse transcription kit (Takara) according to the manufacturer’s instructions. Quantitative PCR was performed using a fluorescence-based PCR system under the following cycling conditions: 95 °C for 30 s, followed by 45 cycles of 95 °C for 20 s, 60 °C for 20 s, and 72 °C for 30 s. Gene expression levels were analyzed via the 2–ΔΔCq method. The primer sequences (Qingke Biotechnology) used are listed in Table 1.

**Table 1:** Gene primers

Gene	Gene sequence (5^/^-3^/^)
KLF4	Forward: CGG​ACC​TAC​TTA​CTC​GCC​TT
	Reverse: CCT​GAA​CCC​CAA​AGT​CAA​CG
β-catenin	Forward: GCC​GGC​TATT​GT​AGA​AGC​TG
	Reverse: GTC​CCA​AGG​AGA​CCT​TCC​ATC
Cyclin D1	Forward: GAA​GGA​GAC​CAT​CCC​CCT​GA
	Reverse: CAA​TGA​AAT​CGT​GCG​GGG​TC
E-Cadherin	Forward: CTT​TGA​CGC​CGA​GAG​CTA​CA
	Reverse: TTT​GAA​TCG​GGT​GTC​GAG​GG
N-Cadherin	Forward: CAT​CCA​GAC​CGA​CCC​AAA​CA
	Reverse: ACA​GAC​ACG​GTT​GCA​GTT​GA
β-actin	Forward: GCC​GGC​TATT​GT​AGA​AGC​TG
	Reverse: GTC​CCA​AGG​AGA​CCT​TCC​ATC

*Western Blot*. Proteins were extracted by lysing cells in RIPA buffer containing PMSF (Solarbio) for 30 min. Following centrifugation (15,000×*g*, 10 min, 4 °C), the supernatant was collected and quantified (Solarbio kit). Proteins were separated via 10 % SDS-PAGE and transferred to PVDF membranes (Millipore). After blocking with 5 % skim milk (2 h, RT), membranes were incubated with primary antibodies (overnight, 4 °C) against KLF4 (1:1,000), β-catenin (1:8,000), Cyclin D1 (1:5,000), E-cadherin (1:1,000), N-cadherin (1:1,000), β-Tubulin (1:5,000), and GAPDH (1:5,000). Subsequently, membranes were incubated with a secondary antibody (1 h, RT, 1:5,000). Signals were developed with ECL reagent (Solarbio), captured using Image Lab™ software (BioRad), and analyzed with ImageJ.

*Statistical analysis*. All data were analyzed with GraphPad Prism 5.01 (Dotmatics) and are presented as the mean ± standard deviation. Comparisons between two groups were performed using the Student’s *t*-test, while the Chi-square test was used for categorical data. Correlations were assessed by Spearman’s rank correlation analysis. A p-value of less than 0.05 was considered statistically significant.

## 3 Results

**Figure 1:**
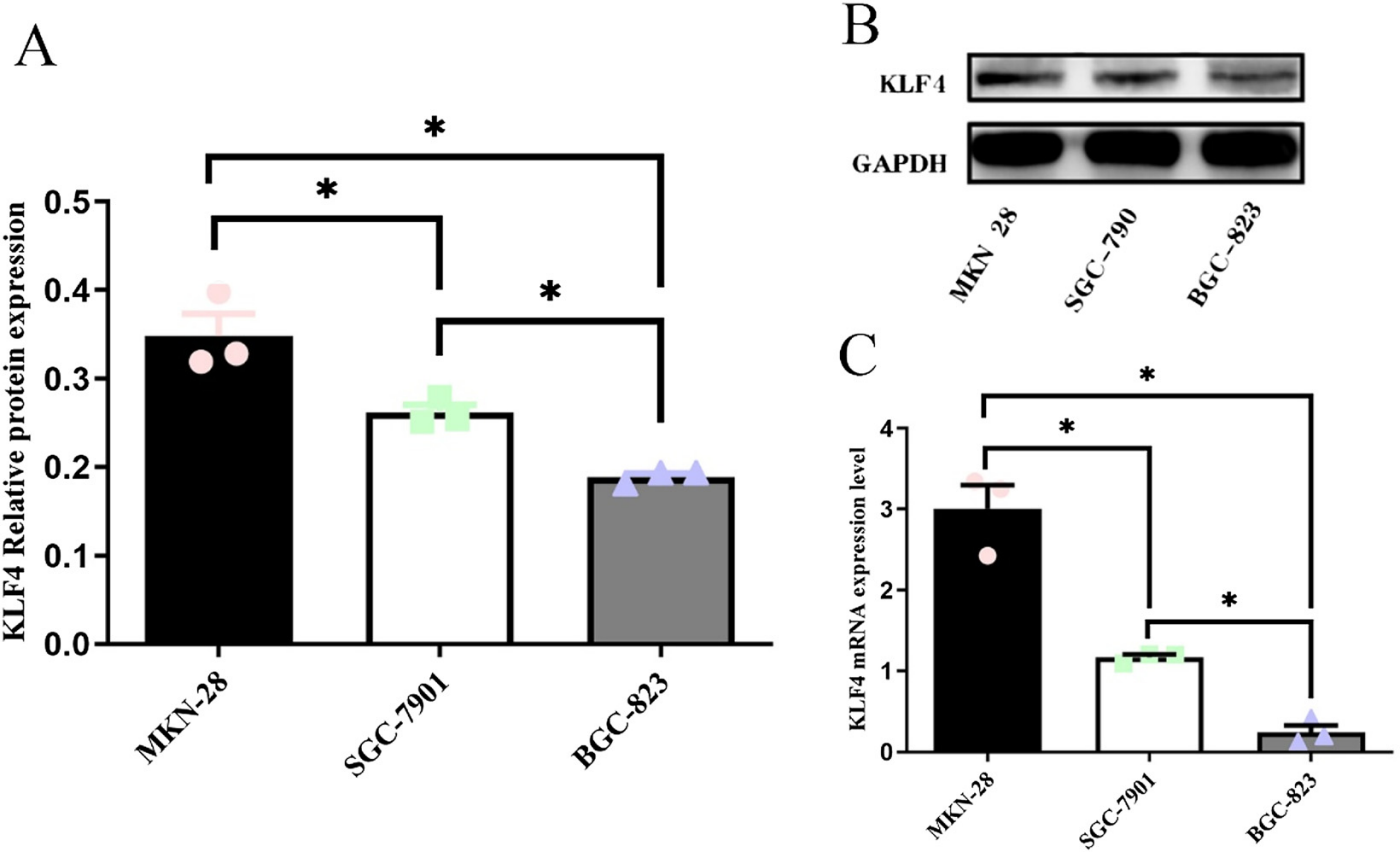
Expression levels of KLF4mRNA and protein in three strains of gastric cancer cells. (A) Histogram of relative mRNA expression levels of KLF4 in the three gastric cancer cells. (B) Protein expression level of KLF4 in three gastric cancer cells. (C) Histogram of relative protein expression of KLF4 in three gastric cancer cell lines. Three individual experiments with at least three replicates were performed. *p < 0.05, three strains of gastric cancer cells were compared two by two separately.

*The expression levels of KLF4 mRNA and protein were quantified in gastric cancer cell lines with varying differentiation degrees*. Both KLF4 mRNA and protein showed a gradual reduction from the well-differentiated MKN-28 cells to the moderately differentiated SGC-7901 and poorly differentiated BGC-823 cells, with statistically significant differences (*p* < 0.05; Figure 1). Spearman’s correlation analysis confirmed a strong positive correlation between the expression levels of both KLF4 mRNA (*r* = 0.949, *p* < 0.05) and protein (*r* = 0.949, *p* < 0.05) with the differentiation degree of the gastric cancer cells.

**Figure 2:**
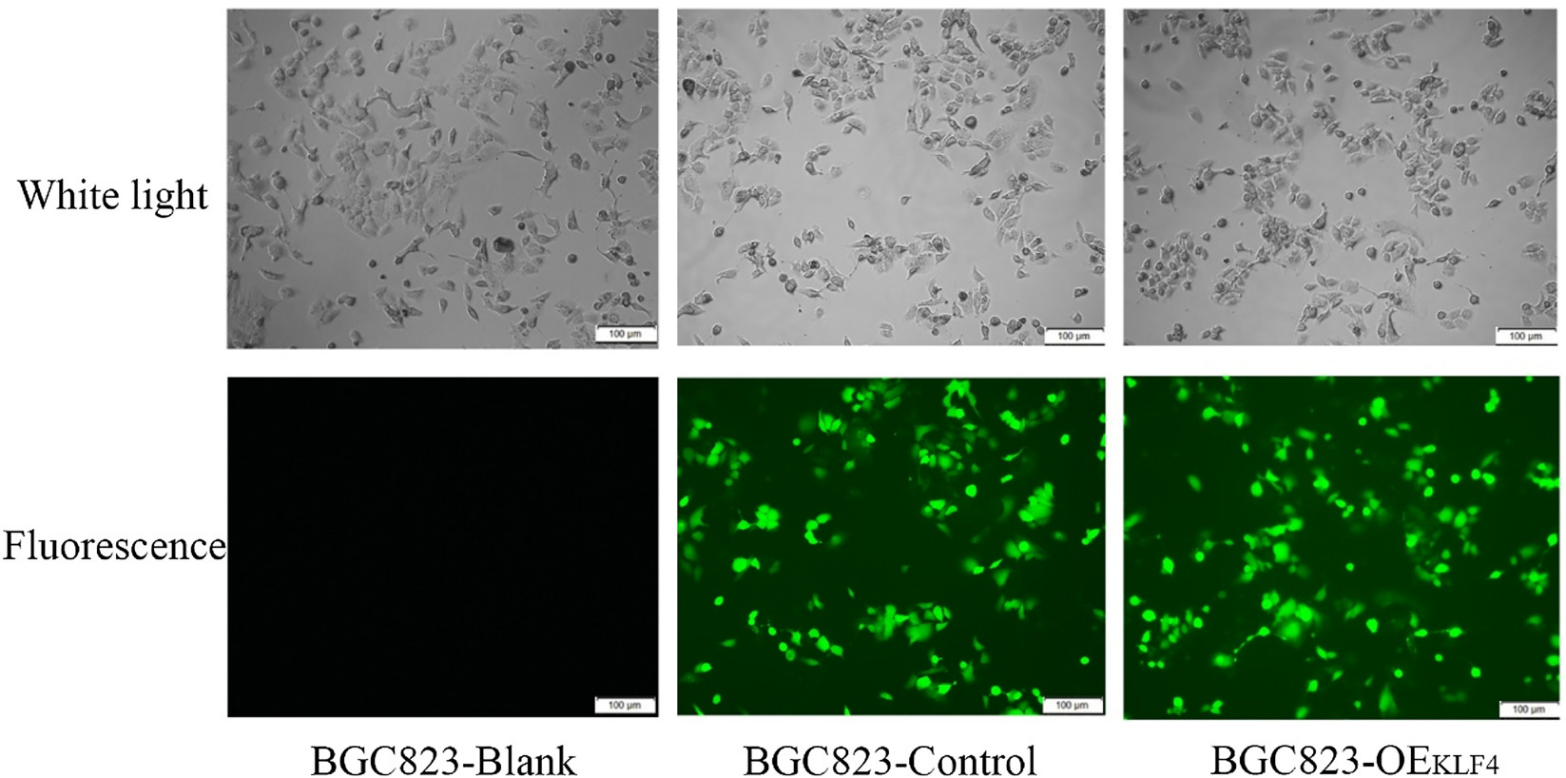
Fluorescence and white light images of each group of cells under inverted fluorescence microscope.

*Construction of KLF4 overexpressed cell line*. To investigate KLF4’s function, we established an overexpression model in BGC-823 cells, which exhibited the lowest endogenous KLF4 levels. Transfection efficiency was confirmed by the presence of green fluorescence in the BGC823-Control and BGC823-OE_KLF4_ groups, but not in the non-transfected BGC823-Blank group, demonstrating successful lentiviral delivery (Figure 2).

**Figure 3:**
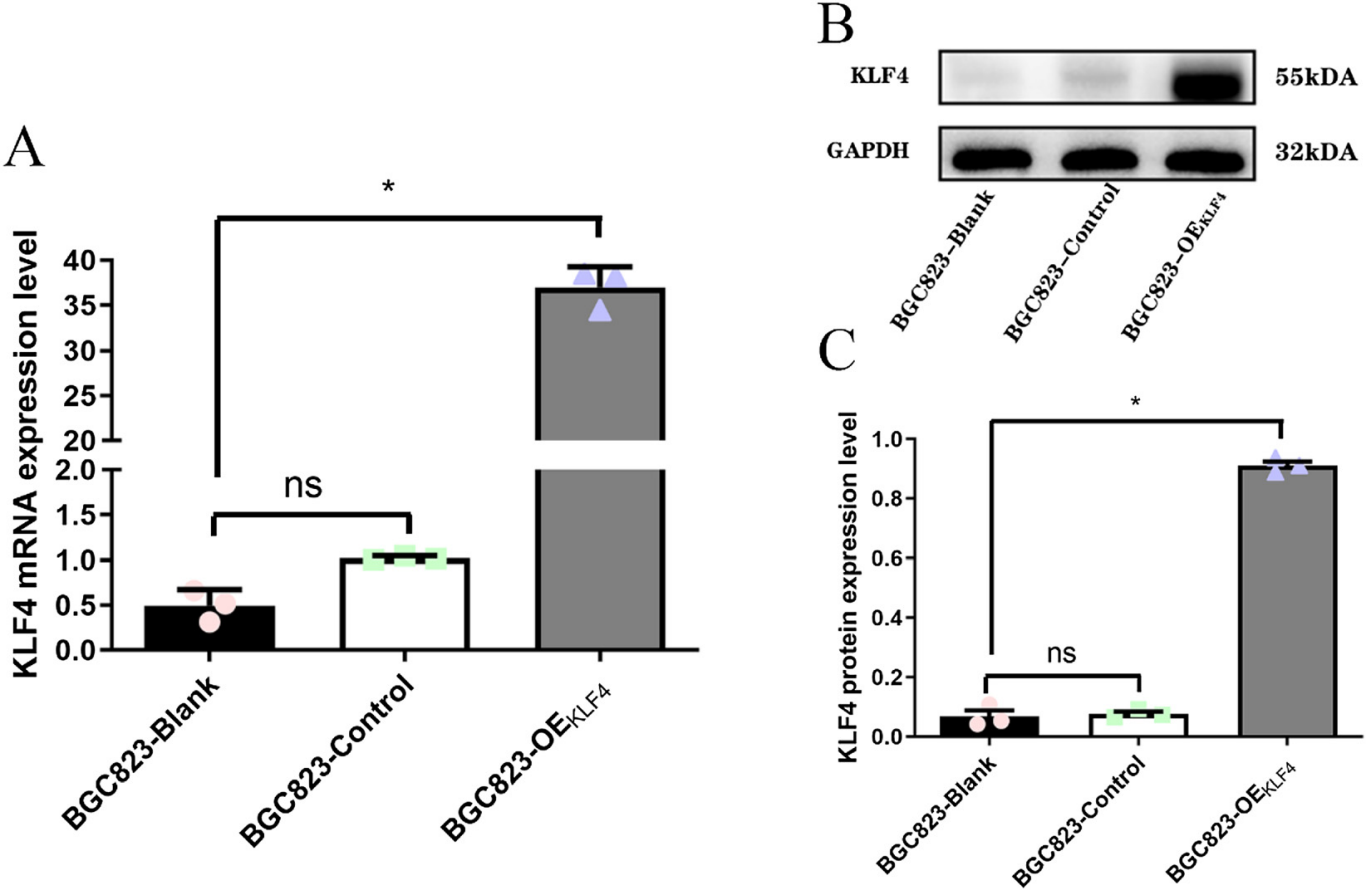
Expression levels of KLF4 protein and mRNA in BGC-823 cells after transfection of KLF4 gene. (A) Histogram of relative expression levels of KLF4 mRNA in three groups of cells. (B) Expression levels of KLF4 protein in three groups of cells. (C) Histogram of relative expression of KLF4 protein in three groups of cells. *p < 0.05, BGC823-OEKLF4 group versus BGC823-Control group. ns, BGC823-Blank group versus BGC823-Control group. ns, no significant.

*KLF4 protein and mRNA expression levels in BGC-823 cells post-transfection with KLF4 gene*. Successful KLF4 overexpression was achieved in BGC-823 cells, as evidenced by significantly higher levels of KLF4 mRNA and protein in the BGC823-OEKLF4 group compared to both the BGC823-Blank and BGC823-Control groups (*p* < 0.05), validating the efficacy of the lentiviral transduction (Figure 3).

**Figure 4:**
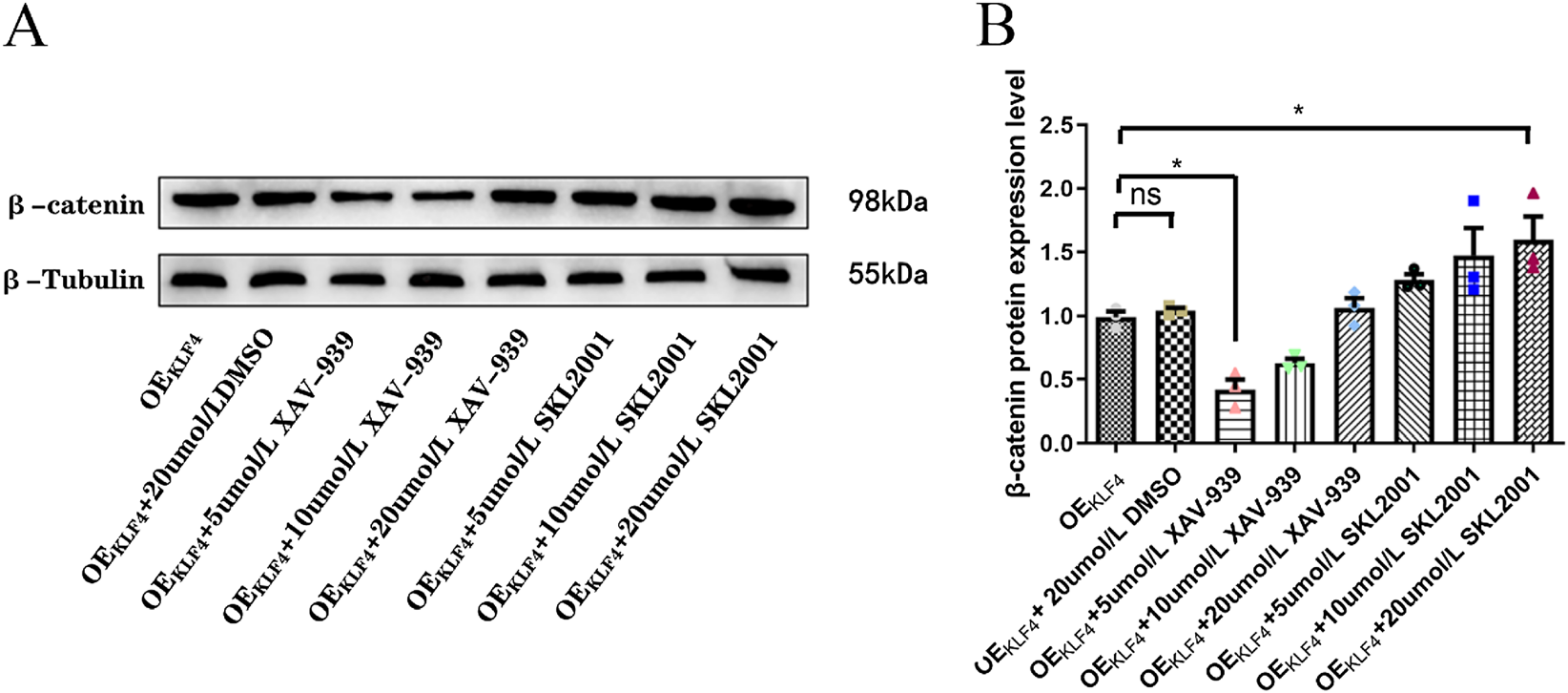
Results after addition of XAV-939 and SKL-2001. (A) β-catenin protein expression level. (B) Histogram of the relative expression of β-catenin protein. *p < 0.05, OEKLF4 group versus 5 μmol/L XAV-939 group, OEKLF4 group versus 20 μmol/L SKL-2001 group. ns, OEKLF4 group versus OEKLF4+20 μmol/L DMSO group. ns, no significant.

*Role of the Wnt/β-catenin pathway in KLF4-mediated EMT regulation using agonist and inhibitor treatments*. The effective concentrations of the Wnt/β-catenin pathway modulators were first determined. It was found that β-catenin was most effectively suppressed by 5 μM XAV-939 and activated by 20 μM SKL2001 in KLF4-overexpressing BGC-823 cells (*p* < 0.05; Figure 4). Furthermore, the potential cytotoxic interference of the solvent DMSO was investigated. A control group treated with 20 μM DMSO, the highest concentration used, showed β-catenin expression levels comparable to the OEKLF4 group alone (*p* > 0.05), confirming that the observed effects were devoid of solvent-related artifacts.

*KLF4 inhibition of EMT in gastric cancer cells via the Wnt/β-Catenin signaling pathway*. We observed a concomitant reduction in the expression of Wnt/β-catenin components (Cyclin D1, β-catenin) and an induction of the epithelial marker E-cadherin in KLF4-overexpressing cells (*p* < 0.05). These findings indicate that KLF4 suppresses EMT in BGC-823 cells, likely by inhibiting the Wnt/β-catenin pathway.

**Figure 5:**
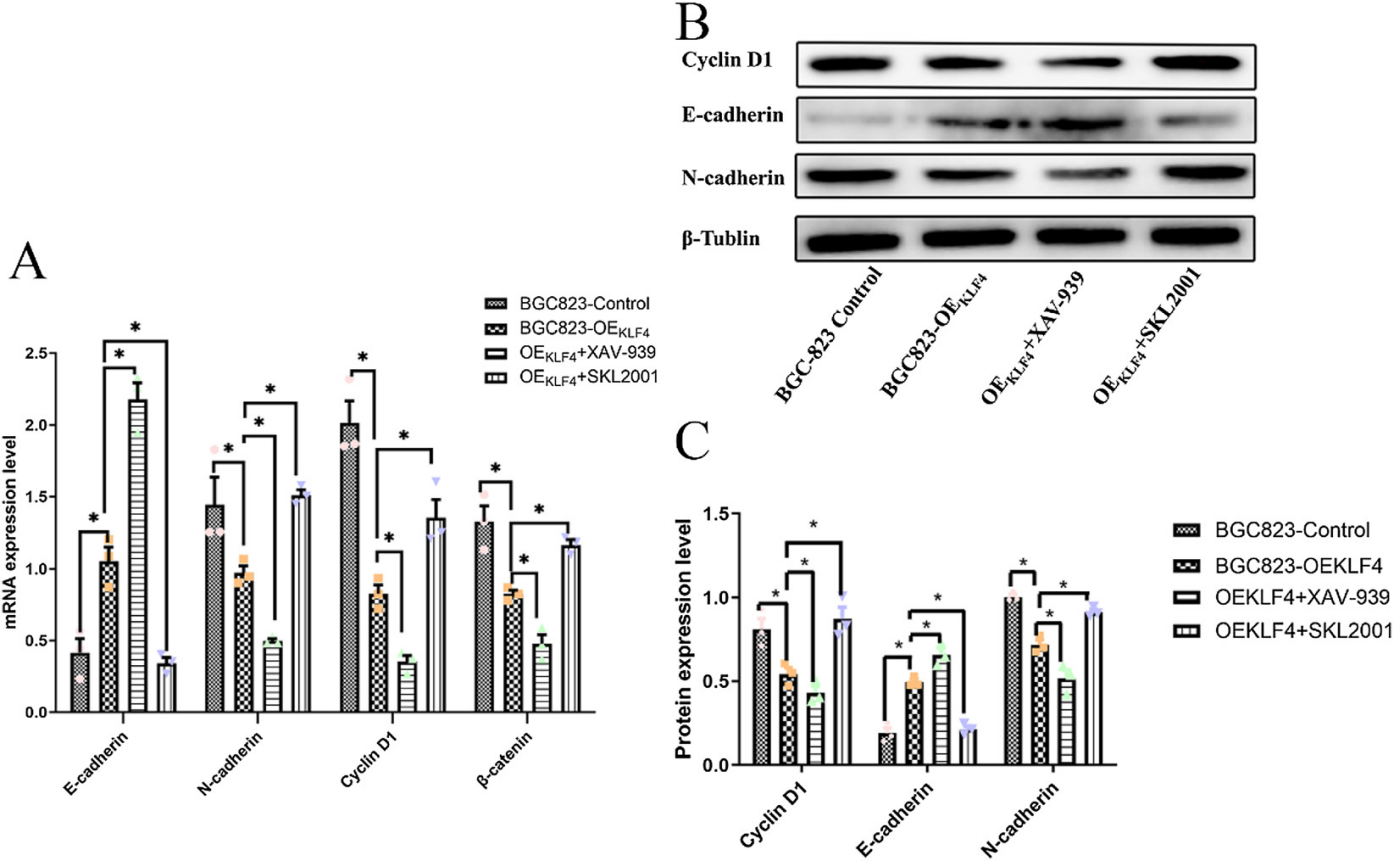
EMT changes after agonism and inhibition of the Wnt/β-catenin signaling pathway. (A) Histogram of mRNA expression levels of Wnt signaling pathway markers and EMT markers in overexpressing KLF4 BGC-823 cells after addition of XAV-939 and SKL 2001. (B) Protein expression levels of Wnt signaling pathway markers and EMT markers in overexpressing KLF4 BGC-823 cells after addition of XAV-939 and SKL 2001 and (C) relative expression histograms. *p < 0.05.

To functionally test this mechanism, we employed the Wnt/β-catenin inhibitor XAV-939 and agonist SKL2001. As expected, the inhibitor XAV-939 further reduced Cyclin D1 expression in KLF4-overexpressing cells, while the agonist SKL2001 restored it (*p* < 0.05), confirming effective pathway modulation. Consequently, XAV-939 treatment promoted an epithelial state by increasing E-cadherin and decreasing N-cadherin. Conversely, SKL2001 treatment reversed this effect, driving a mesenchymal phenotype with decreased E-cadherin and increased N-cadherin (*p* < 0.05; Figure 5). These rescue experiments demonstrate that KLF4 may inhibits EMT in BGC-823 cells specifically through the Wnt/β-catenin pathway.

## 4 Discussion

Globally, gastric cancer represents the fifth most frequently diagnosed cancer (approximately 6 % of all cases) and is responsible for the third highest number of cancer-associated fatalities [24]. KLF4, a member of the Krüppel-like factor (KLF) family, is a zinc-finger transcription factor predominantly expressed in terminally differentiated epithelial tissues, including the skin, lungs, and gastrointestinal tract. It exhibits a dual role in tumorigenesis, acting as either an oncogene or a tumor suppressor, and participates in diverse molecular pathways and cellular processes [25]. Although the downregulation of KLF4 in gastric cancer has been well-documented and is strongly associated with disease progression, the precise molecular mechanisms through which KLF4 influences cancer cell metastasis remain unclear [24], [25], [26], [27]. EMT is fundamentally linked to cancer metastasis, as evidenced in numerous tumours including those of the breast, ovary, and colorectum [28], [29], [30]. In hepatocellular carcinoma, low KLF4 expression predicts poorer overall and recurrence-free survival [10]. We therefore hypothesized that KLF4 expression might be linked to the differentiation status of gastric cancer. We subsequently observed a clear positive correlation between the two. In addition, while our previous work confirmed that KLF4 overexpression inhibits gastric cancer cell proliferation and migration, the mechanistic basis for this effect remained unclear [31]. Given the unclear mechanism, this study prioritized elucidating the underlying pathways. We focused on the Wnt signaling pathway, given its critical roles in embryonic development and physiology, and its well-established dysregulation in disease, particularly cancer [32]. Wnt signaling pathways are broadly categorized into two types: the canonical, β-catenin-dependent pathway and the non-canonical, β-catenin-independent pathway [33]. The Wnt/β-catenin signaling pathway is well-established as a key regulator of EMT [34], 35], although the underlying mechanisms require further investigation. KLF4, the Wnt/β-catenin pathway, and type 1 EMT are all inextricably linked to embryogenesis and organogenesis. Conversely, dysregulation of KLF4, disruption of Wnt/β-catenin signaling, and type 3 EMT are associated with tumorigenesis and a range of diseases [36], [37], [38]. Based on these findings, we investigated the relationship among these three factors. Our results demonstrated that KLF4 overexpression inhibited both the Wnt/β-catenin signaling pathway and EMT. This suggests that KLF4 may suppress EMT in gastric cancer cells by inhibiting the Wnt/β-catenin pathway; however, the precise mechanism underlying this effect requires further investigation. A limitation of this study is the use of a single gastric cancer cell line. Therefore, future research should be extended to include multiple cell models to validate these findings.

The Wnt pathway is modulated by agonists, which promote signaling to drive cell growth and development, and inhibitors, which attenuate signaling to curb aberrant proliferation and tumor development [39], 40]. The precise modulation of the Wnt pathway holds significant therapeutic promise. This is exemplified by XAV-939, a potent tankyrase (TNKS1/2) inhibitor that suppresses Wnt/β-catenin signaling by stabilizing AXIN and facilitating β-catenin degradation [41]. SKL2001 functions by disrupting the Axin/β-catenin interaction, thereby leading to the stabilization of intracellular β-catenin [42]. In this study, we observed that the inhibitory effect of XAV939 on β-catenin was attenuated at higher concentrations. This paradoxical effect may be attributed to the distinct roles of Axin1 and Axin2, both of which are regulated by TNKS1/2. Specifically, Axin2 is a known target gene of Wnt/β-catenin signaling and functions as part of a negative feedback loop. We hypothesize that at elevated concentrations, the compensatory induction of Axin2 via this feedback mechanism may counteract the inhibitor’s intended effect [43]. Consequently, at high concentrations, TNKS1/2 inhibitors may over-activate the Axin2-mediated negative feedback loop, thereby attenuating the suppressive effect of XAV-939 on β-catenin. However, this remains a preliminary hypothesis. The observed effects could be due to drug toxicity or off-target effects. The specific underlying mechanism requires further investigation.

In summary, our work reveals novel aspects of KLF4-regulated EMT signaling in gastric cancer, providing a foundation for new therapeutic strategies. It also highlights the need to further decipher the precise mechanism of XAV-939’s action on the Wnt/β-catenin pathway. Investigating these deeper mechanisms sets the stage for our future research.
